# The Therapeutic Value of Alcohol1Read before the Buffalo Medical and Surgical Association, November 12, 1889.

**Published:** 1890-01

**Authors:** J. W. Grosvenor

**Affiliations:** 118 Plymouth Avenue


### THE THERAPEUTIC VALUE OF ALCOHOL[1].

By J. W. GROSVENOR, M. D.

Who the discoverer of alcohol was, authentic history does not inform us. We are even ignorant of the name of the person who invented the art of distillation. It may have had its origin in China, or India, or Arabia. There is some ground for believing that Avicenna, a Moorish physician, who died in 1036, had a knowledge of distillation, and used this knowledge in his medical practice. History affords indisputable evidence that Albucasis, a distinguished Moorish physician and chemist who died in the year 1106, was acquainted with the art of distillation, and made brandy.

The term alcohol, as a name for the spirit distilled from wine, did not come into use until the sixteenth century. Since that date, the

1. Read before the Buffalo Medical and Surgical Association, November 12, 1889.

word has obtained a much wider application. Chemical science, in the last fifty years, has evolved many allied substances of different chemical composition named alcohols. Unless otherwise indicated, alcohol, in this paper, will refer to ethyl alcohol—that kind which is in common use for internal administration.

The early history of alcohol, as a therapeutic agent, is enveloped in considerable obscurity. No historical record informs us who first used it as a remedy for disease, nor the time and place of such use. It is reasonable to suppose that the people who first used wine, or other alcoholic liquid, as a beverage, was the first to employ it as a medicine. The exalted position given to wine, and the marvelous virtues attributed to it, by the early nations, would be likely to direct attention to it as a healer of disease. The alchemists regarded the spirits of wine as the elixir of life; from this view, it would only be a step to the suggestion that it would ward off death.

The first work upon the use of alcohol as a medicine, entitled "Concerning the Use and Utility of Brandy," was written by a German physician, Dr. Michel Schrick, and published in 1483. By the close of the sixteenth century, the use of alcohol as a medicine was common among all the nations of Europe, and was regarded, in some quarters, as almost a panacea. Its effect upon mind and body was considered as little less than marvelous. Here and there, along the medical career of alcohol, have arisen physicians who have disbelieved its wonderful efficacy. In 1725, was issued a book from the pen of Dr. George Cheyne, an English physician, in which he represents alcohol as scarcely deserving a place in the apothecary's shop. Thomas Beddoes, a talented English physician and chemist, in his Hygeia, published in 1802, called attention to many dangers resulting from the medicinal use of alcoholics. As early as 1832, Dr. John Higginbotham, of Nottingham, Eng., had abandoned the medical prescription of alcohol. He was a man of strong mind, and wrote several articles on the non-alcoholic treatment of disease, which were published in the London *Lancet* and *British Medical Journal.* An ardent advocate of the medical use of alcoholics appeared in the person of Robert Bentley Todd, a London physician and noted physiologist, who flourished in the middle of the present century, and wrote "Clinical Lectures on Certain Acute Diseases." He specially favored the use of alcoholic medication in fevers. His argument was, that these diseases were weakening in their nature, and needed supporting treatment through all their stages; that alcohol was a food, and was capable of furnishing, to the debilitated system, the materials needed for its support. Dr. Todd was a vigorous and voluminous writer, and secured the following of many physicians of his time. The author of this paper recalls the treatment of typhoid fever, twenty-five years ago, by Dr. A. L. Loomis, in the wards of Bellevue Hospital, New York City. His favorite remedy at that time was whiskey, of which three pints, or two quarts, for a patient each twenty-four hours, was not an uncommon quantity.

Soon after the death of Dr. Todd, above mentioned, there was a change in medical opinion concerning the efficacy of his excessive alcoholic treatment. Many of his imitators largely reduced their doses of alcohol, and there started a flow of opinion in favor of non-alcoholic medication in all diseases. In 1866, F. R. Lees, an English physician, published his book, "Is Alcohol a Medicine?" This work awakened thought and stimulated investigation. A few years of agitation resulted in the establishment of the London Temperance Hospital, the *Medical Temperance Journal*, and the British Medical Temperance Association, for the purpose of investigating the medicinal virtues of alcohol, and of testing the utility of treating disease without alcohol.

In this country, Dr. N. S. Davis has occupied a prominent position for the past twenty-five years, in favor of an extremely limited use of alcoholic medication. At the twenty-third annual meeting of the American Medical Association, in May, 1872, "a resolution to discourage the use of alcohol in medicine was unanimously carried." At the meeting of the International Medical Congress, held in Philadelphia, in September, 1876, Dr. Ezra M. Hunt, of New Jersey, presented to the Medical Section a paper on the subject of Alcohol as a Food and Medicine. Dr. Hunt took the position that the field for the action of alcohol as a proper medicinal agent was exceedingly narrow. The sentiments of this paper were quite unanimously agreed to by the Section on Medicine.

It is believed that, during the last two decades, a tide of medical opinion has been setting towards a restricted use of alcohol as a remedial agent in disease. This is notably the case in England. Sir William Gull, Sir Henry Thompson, Drs. Richardson, Ridge, Kerr, Ritchie, Edmunds, Munroe, Parkes, and Hare, all well-known English physicians, may be reckoned on the side of medical reform in this respect. Other countries of Europe furnish an occasional bright name, of which the same statement may be truly made. In this land, Drs. Davis, Hunt, Hargreaves, Story, and many lesser lights, are earnest advocates of confining alcohol, as a medicine, within very narrow limits.

Doubtless, the majority of physicians of to-day prescribe alcohol with the same freedom as did the medical profession of twenty-five years ago; quite a large minority use much more discrimination in this regard; a comparative few use alcohol, remedially, in only rare instances; here and there may be found a medical practitioner who has altogether discarded it from his list of remedial agents. It is my belief that the medical profession of the world is losing its faith in the beneficial effects of alcohol upon the diseased human system. As the day of indiscriminate phlebotomy has passed away, so is passing away the inconsiderate exhibition of alcoholic medicines.

It is a fact, more clearly and more universally recognized than formerly, that the medicinal ingredient of all alcoholic liquids is alcohol. It is the alcohol in beer, wine, whiskey, and brandy, upon which the physician depends for his desired effect, except that beer may sometimes be prescribed on account of the hops it contains. If these alcoholics were deprived of their alcohol, their special virtue would disappear, and no physician would recommend them to his patients.

The value of alcohol as a therapeutic agent can only be determined after a due consideration of its characteristics and its effects upon the human system.

Alcohol is a poison. This characteristic has been clearly recognized by eminent chemists, physiologists, and toxicologists of this country and of foreign lands. Several medical dispensatories and medical dictionaries have pronounced it a poison. This has been the declaration of such men as Taylor, Copeland, Christison, Regnault, Pereira, Carpenter, Orfila, Beck, and Mussey. This truth has been demonstrated upon the lower animals as well as upon man. Dr. Percy injected two and a half ounces of alcohol into the stomach of a full-grown dog. It fell lifeless to the ground at once. Six ounces of alcohol being thrown into the stomach of a large dog, by the same experimenter, it fell at once and never afterward moved a limb, ceasing to breathe after an hour and twenty minutes. Fontane performed similar experiments upon frogs and turtles, with like results. The experiments of Mitscherlich upon rabbits, and of Jacobi and of Falck upon pigeons, rabbits and dogs, arrived at similar conclusions.

When introduced into the human stomach, in large doses, alcohol produces the same poisonous effect as upon animals. Prof. Christison mentions the case of a man who stole a bottle of whiskey and died in four hours after drinking it. Dr. Mitchell in his Therapeutics cites the case of a boy, ten years of age, who drank from his father's whiskey bottle and was dead in less than an hour. Orfila in his Traité de Toxicologie refers to the case of a man who died immediately on taking a large dose of brandy. In his Medical Jurisprudence, Dr. Taylor tells of a man who drank a bottle of gin for a wager, and died in half an hour. Dr. P. DeMarmon, of King's Bridge, New York, has reported the case of a boy, five years old, who died in nineteen hours after drinking a glass of whiskey. Into the Northern Hospital, Liverpool, Eng., in the year 1868, were admitted thirty-six cases of acute alcoholic poisoning. Doubtless, a similar statement might be made concerning every large general hospital in Europe and in this country. Probably but few physicians have practised five or ten years who have not seen several such cases, and many city physicians have come in contact with scores of them.

In cases of sudden death from large quantities of alcohol, doubtless, a profound effect has been made upon some of the great nervous centers. Alcohol, however, finds its way so rapidly into the various organs that even, in many of these cases, a poisonous effect is produced upon the general system. In cases of ingestion of alcohol in small quantities, over long periods of time, its effect is equally poisonous and death is equally certain, as in cases of large doses taken at once, as mentioned above.

Dahlstrom experimented for a period of eight months upon three dogs, and daily gave each of them six ounces of potato brandy. At the end of this time one died and two were killed. The undoubtedly poisonous effects of alcohol were shown all along these eight months of experimentation. Duchek made similar experiments upon dogs, with like results. The many cases of chronic alcoholism seen by every physician, afford indubitable proof of the poisonous effects of alcohol on the human organism, even when taken in moderate amounts if long continued.

The question whether alcohol is a food has been discussed during the past fifty years with considerable energy and thoughtfulness. Both sides of the question have had able advocates. It has been held that in tissue-wasting, and debilitated conditions of disease, alcohol is useful as a food-support to the system. Foods have been divided into two general classes, nitrogenous and carbonaceous, the former being the tissue-making, the latter the heat-producing foods. This classification may not be strictly correct, under all circumstances; for food, which, under ordinary conditions, produces tissue, may, under exceptional conditions, produce heat, and *vice versa*, but this division may be sufficiently accurate for the purposes of this essay.

Alcohol contains no nitrogen and none of the compounds which belong to tissue-making foods; its action upon the system does not accord with that of the usual nitrogenous foods. Hence, there is no solid ground for believing it to be a nitrogenous food; indeed, the advocates for regarding it in this light are exceedingly few. If alcohol is a carbonaceous food, its behavior should be similar to articles which are clearly recognized as carbonaceous foods. The experiments of Dr. N. S. Davis conclusively prove that during the digestion of all kinds of food the bodily temperature is increased, while, within half an hour after the ingestion of alcohol, the temperature falls and continues to be lowered for the coming two or three hours. Dr. B. W. Richardson's observations are in accord with those of Dr. Davis. The paralyzing effect of alcohol upon the vaso-motor system of nerves weakens the contractile power of the minute blood-vessels; an unusual quantity of blood reaches the surface of the body, and thus, for a brief period, elevates its temperature.

Other experimenters have noted the same or similar effects. If alcohol is a heat-producer, it ought to undergo oxidation, and the products of its oxidation, which are aldehyde and acetic acid, should be discoverable. These articles have not been found in the system, or they have appeared in such an almost infinitesimal quantity, that their presence is scarcely an argument in favor of the heat-producing nature of alcohol.

The heat-value of the carbonaceous foods is largely determined by the amount of carbonic acid exhaled from the lungs during their digestion. The richer in heat-quality is any food the larger amount of carbonic acid exhaled will follow its ingestion. No increase in the quantity of carbonic acid thrown off from the lungs, follows the ingestion of alcohol—in fact, a decided decrease has been noted by some experimenters. In a series of experiments made three-quarters of a century ago, Dr. Prout, according to the statement of another, "established the fact that alcoholic liquors diminished the quantity of carbonic acid eliminated more than any other article he experimented with." In Dr. Prout's own words, "alcohol enormously depresses the combustion of the carbon of the system during its existence in the body."

Anstie, a strong advocate of the usefulness of alcohol as a medicine, on the ground that it produces some kind of force in the system, declares that "this force cannot be heat." It is the uniform experience of those subjected to the rigors of northern latitudes, that alcoholic beverages do not increase the power of resistance against intense cold. Total abstainers have best withstood the hardships of Arctic expeditions.

It is generally admitted that force accompanies the production of heat, and that without heat there is no force. Granting this to be true, and that alcohol has no heat-producing power in the body, of what utility is it as a force-generating food?

It has been claimed that the extra accumulation of fat by habitual drinkers of alcoholics is an evidence that alcohol is a food. It has not been proven that such an accumulation exists in cases of those who drink pure alcoholics. If such a condition exists at all, it is due to articles such as sugar taken in combination with the alcohol. Such a condition may not contribute to the health of the body. It is a fact, acknowledged by all who are conversant with this subject, that alcohol produces fatty degeneration in various organs and tissues of the body. It has been aptly termed the "genius of degeneration." It is hardly appropriate to call that article food which so largely contributes to disease as alcohol. It has been maintained that alcohol, if not a tissue-forming nor a heat-producing food, is a food in the sense that it retards tissue waste. Experiments to ascertain whether the waste of tissue is less rapid under the use of alcohol than without it, have been made by Dr. E. A. Parkes. He regarded the elimination of urea as a fair test of the amount of tissue wasted. His experiments were made upon a healthy soldier. Notice was taken of the amount of urea excreted when at work and drinking simple water, and also when at work and drinking twelve ounces of brandy each day. The amount varied so little under the two conditions that he very properly concluded that alcohol had no effect in retarding the waste of tissue. Accepting this conclusion as correct, alcohol cannot be regarded as a food in the sense that it retards regressive metamorphosis.

If alcohol does not promote the formation of tissue, does not increase the heat of the body, and fails to prevent any waste of tissue, it is difficult to see in what sense it should be called a food. If not a food, there is no reason for administering it in disease on the ground that as food it will support the system.

The question whether alcohol is a stimulant has been the subject of sharp controversy. Since its discovery, until a comparatively brief period, the belief that it was a genuine stimulant reigned supreme among all classes. Much of this difference of opinion has hinged upon the difference of definitions given to the word stimulant. If a stimulant is something which adds force to the system, it is doubtful if alcohol can be properly styled a stimulant. If a stimulant is something which irritates and excites the system, without bestowing upon it any power, alcohol may lay some claim to the term stimulant. The most casual observer may notice that a person who has taken alcohol in some form is in an excited condition, physically and mentally. The excitement lasts a longer or shorter time, depending upon the amount of alcohol taken. If the quantity taken is large, there is either no stage of excitement or this soon passes away to be followed by a stage of depression. It is my belief that whatever excitement may follow a dose of alcohol, is produced by its depressing character. The vaso-motor nerves become partially paralyzed, an unusual quantity of blood is collected on the surface of the brain and other nervous centers, mental processes move more quickly, physical acts become more rapid, and thus is produced that systemic condition usually denominated stimulation. Ere long, the nerve centers may become so engorged with blood, that from pressure and from the poisoned condition of the blood, partial or complete paralysis will supervene.

If this explanation is correct, the excitement has, as its cause, the depressing nature of the alcohol. The alcohol has produced the excitement, or stimulation, through a depressing act. Is it not appropriately styled a depressant, rather than a stimulant? It may be that a portion of the excitement is produced by the irritating force of alcohol upon all the tissues with which it comes in contact.

It is a clearly demonstrated fact that a portion, at least, of the alcohol injected circulates through the system, and is excreted without undergoing any change. The experiments of Dr. J. J. Ridge have shown that, under a very moderate dose of alcohol, keenness of vision is lessened, hearing power is diminished, and the sense of touch becomes less acute. Dr. Brinton says: "Even a moderate dose of beer, or wine, diminishes the maximum weight which a person can lift to something below his teetotal standard." Dr. T. K. Chambers, in defining a stimulant, says: "It is usually held to be something which spurs on an animal operated upon to more vigorous performance of its duties. It seems doubtful if, on the healthy nervous system, this is ever the effect of alcohol, even in the most moderate dose, and for the shortest period of time." Dr. Anstie regarded alcohol as a stimulant in small doses, and in large doses a narcotic. It is the belief of some eminent physicians that, under no circumstances, does alcohol produce the effect of stimulation. Dr. Edmunds declares: "The so-called stimulating effects of alcohol are really only finer shades of that same narcotic influence which produces general stupefaction and universal paralysis when the agent is given in large doses." Alcohol has often been likened to a spur, which calls forth the strength of the horse, but adds nothing to his powers of endurance; indeed, it sooner or later brings on exhaustion.

In determining whether alcohol shall be called a narcotic or stimulant, is it not better to have regard to its inherent quality of narcotism, rather than to a doubtful stimulating effect which it may produce? My belief is that its mode of action is always that of a narcotic, and hence, I prefer to call it a narcotic.

Alcohol has been called an antispasmodic. If it possesses this characteristic, it doubtless arises from its narcotic property, whereby it quiets the nervous centers.

Alcohol is an antiseptic. It prevents the combination of oxygen with dead animal substances, and thus acts antiseptically. Doubtless it acts in the same way in the living body. Experiments of Dr. J. J. Ridge show that, under some circumstances, alcohol acts as a septic agent. Very small percentages of alcohol favor the development of bacteria. In simple water, and in water containing one per cent. of alcohol, bacteria multiplied at about the same rate. More than one per cent. retarded, while less than one per cent. favored their growth. One quarter to one-half per cent. was the quantity most favorable to their development. His experiments were tried with infusion of hay. The conclusion of this experimenter was, "that the alcohol must act as a stimulant to the vital reproductive functions of these minute organisms, which, undoubtedly, contain a certain special kind of protoplasm." His experiments on cress-seed, and the young cress-plant, declared that no quantity, however small, stimulated the growth of their protoplasm. In the experimenter's own words: "We may say, then, that alcohol injures constructive protoplasm and stimulates destructive protoplasm. In other words, alcohol hinders construction and promotes destruction."

As an anesthetic, alcohol has been effectively used in combination with other anesthetics.

Alcohol is an antipyretic. It reduces bodily temperature, as has many times been proven by the experiments of eminent physiologists and chemists. No one has done more faithful work in this direction than Dr. B. W. Richardson. His experiments covered a period of three years, and were made upon both man and the lower animals. According to his observations, the first effect of alcohol was a rise of external temperature, sometimes to ½° F., and, in the confirmed inebriate, to 1½° F. This effect is, in fact, a process of cooling. An unusual quantity of blood is thrown to the external surface, through the paralysis of the vaso-motor nerves, and thus is started an unwonted rapidity of radiation of heat. In a brief period of time—say in half an hour—the temperature commenced to fall, and continued to decline for two or three hours, generally reaching 2½° or 3° F., and sometimes 8° below the normal point. The amount of time necessary for the return of the full amount of natural heat was generally seven or eight hours, and sometimes a period of three days. The quantity of alcohol taken to produce these effects was sufficient to accomplish complete intoxication. It was observed that the extent of reduction of temperature was in direct proportion to the quantity of alcohol taken. The experiments of Dr. N. S. Davis, which commenced in 1850, arrived at results almost identical with those above mentioned. Dr. Franz Riegel has shown that even moderate doses of alcohol reduce the temperature some tenths of a degree. Drs. Binz and W. A. Hammond have also shown that there is a lowering of bodily temperature while under the influence of alcohol. A case of alcoholic poisoning is reported in the London *Lancet* of March, 1878, in which the temperature fell to 92°. Doubtless it is the prevailing belief to-day, among those who have studied the influence of alcohol on the human system, that it is a positive antipyretic.

In estimating the value of alcohol as a medicine, it is necessary to take into account its injurious, as well as its beneficial influence. Alcohol exerts a deleterious influence on the blood. Upon the coagulating quality of the blood, it acts in one of two ways: either fixing the water of the blood with its fibrin, and thus diminishing its coagulating power, or extracting the water, and thus causing coagulation too easily. It changes the form and lessens the size of the red corpuscles, thus diminishing their capacity to absorb oxygen and carbonic acid. It also dissolves both the white and red corpuscles. The corpuscles adhere together, and form masses, instead of retaining their normal separation. Blood, under alcoholic influence, contains an abnormally large quantity of fat. One-half per cent. of alcohol in the blood is sufficient to produce profound intoxication. These conclusions are results of experiments and observations made by Richardson, Virchow, Boecker, Dogiel, and others.

The experiments of Dr. Parkes and Count Wallowicz, made for determining the influence of alcohol upon the heart and circulation, showed that eight ounces of alcohol given in twenty-four hours, increased the number of heart-beats one-fifth during that time, and burdened the heart with an amount of extra work, equivalent to lifting twenty-four tons one foot. A few days subsequent to leaving off the alcohol, the heart showed an unusual feebleness. Dr. N. S. Davis has shown, by *post mortem* examination, that the heart strongly influenced by alcohol is subject to fatty degeneration. Dogiel observed that alcohol at first accelerated, and then retarded the heart-beat; that the greatest retardation of the circulation occurred in the stage of alcoholic narcotism.

On the nervous system, alcohol acts as a paralyzer. At first it excites, and then blunts the sensory nerves, so that impressions are ess keenly felt, and are conveyed with less rapidity and accuracy. Dr. Howie declares that a message which can ordinarily be sent from the hand to the brain in .1904 of a second, required .299 of a second to pass over the same route after two glasses of hock had been taken.

For the brain, alcohol seems to have a special affinity. Taken into the stomach, it is soon circulating through the brain as alcohol. If, after the injection of alcohol, the amount obtained from a given weight of blood is represented by 100, the amount obtained from brain tissue is represented by 134. The brain becomes congested, its membranes thickened, its substance hardened, and its functions crippled, or destroyed. Other important organs of the body are deleteriously affected by alcohol. How common is the cirrhotic, or whiskey liver! How frequent that degeneration of kidneys which has its foundation in the use of alcohol! Dr. W. A. Hammond declares that more than a score of nervous diseases are the direct result of alcoholic drinks, besides various forms of insanity. Alcoholic paralysis finds an acknowledged place in our catalogue of diseases. Alcoholic phthisis is claiming recognition as one of the forms of that disease. Hardly an organ or tissue of the habitual drinker of alcoholics escapes the deteriorating influence of alcohol.

Let us turn our attention to the applicability, or the reverse, of alcohol in certain diseased conditions, for which it has been recommended and used, keeping in mind its characteristics and its harmful influences, already mentioned.

In sudden feebleness of the circulation, whether due to heart failure, nervous shock, or some other cause, the exciting power of alcohol in small and frequently repeated doses has a utility. Thus used, it does not appear to have any advantage over other special excitants, such as heat, ammonia, ginger, capsicum, camphor, while its disadvantages are serious.

For indigestion and difficulty of digestion, alcohol has been largely used by the laity and the profession. The fact that alcohol coagulates the pepsine of the gastric juice, hardens the foods, especially albumin, irritates the mucous membrane of the stomach, and degenerates the blood from which the digestive juices are derived, is a contra-indication for its use in these conditions. Alcohol may arouse the flagging energies of the digestive organs for a brief period, and thus increase the quantity of digestive juices, but there is a limit to the powers of nature, and ere long there is only a feeble response to the demands thus made. If the digestive powers are exhausted, how much more reasonable to give them a rest and aid them by the use of partially digested foods, than in their exhausted condition to spur them on to increased work. The day has come when the treatment of disease by nutrients is having, and should have, large consideration by the medical profession. Alcohol being a narcotic and anesthetic, has been found useful in all diseased conditions accompanied by pain, but it is neither so certain nor so prompt in its utility of allaying pain as opium, the ethers, and chloroforms.

Its antipyretic property gives alcohol a utility in all diseased conditions which demand a reduction of temperature. To be of any special service as an antipyretic, it must be used in large doses, and hence it is quite probable that it may be more harmful than useful. At all events we have other antipyretics at our command which are devoid of the injurious qualities of alcohol, and are far superior as antipyretics, such as cold bathing, quinine, antipyrin, antifebrin, and exalgin.

As an antiseptic, alcohol has been used both locally and internally in diphtheria, scarlet fever, and in other diseased conditions calling for antiseptic treatment, but it cannot be regarded as comparable in antiseptic power to our modern antiseptics.

Perhaps in no one disease has alcohol been more frequently and more largely used than in typhoid fever, both for its antipyretic and its alleged stimulating powers. Since the days of Dr. Bentley Todd, who was the champion of the use of alcohol in this fever, its treatment has undergone a marked change. Dr. W. T. Gairdner, physician to the Glasgow Fever Hospital, gives statistics of 595 cases of typhus fever—doubtless referring to the fever now generally denominated typhoid—treated by himself in the year 1863. The death-rate was 11.9 per cent. The death-rate of 1861 and 1862, when Dr. Gairdner was not in attendance, was 17½ per cent. The average mortality from this disease at that time in English hospitals was a little less than 18 per cent. Under Dr. Gairdner's superintendence the average amount of alcoholic spirits was 2½ ounces, and of wine 2¼ ounces, while in the two previous years it was six ounces of spirits and 34 ounces of wine. The mortality of Dr. Todd was twenty-five per cent. The death-rate from typhoid fever in St. George's Hospital, London, from 1877 to 1883, under alcoholic treatment, was twenty-four per cent.

Dr. A. L. Loomis, of New York, who has had an extensive experience with typhoid fever, excludes alcoholics entirely in the milder cases, and in all cases uses them with great care and watchfulness. His method of treatment to-day is in marked contrast with that of twenty-five years ago, when he relied upon whiskey to the amount of from one pint to three pints daily.

All cases of typhoid fever under my care for the last several years, have been treated without alcohol and with very satisfactory results.

Until within a comparatively recent date in the treatment of delirium tremens, the almost uniform medical belief has strongly and tenaciously favored the use of alcoholics. The day has passed for holding a physician liable to censure who advocates a total disuse of alcohol in this disease. For many years it has been my practice to at once and utterly discard it, in even the severest cases, substituting therefor some form of bromide, and proper alimentation. Remove the alcoholic poison from the system by evacuants as quickly as possible and forbid its entrance again! A continuation of the poison prolongs the poisoned condition, and lays the foundation for future disease.

The fact that alcohol paralyzes the vaso-motor nerves, and thus lessens the contractile force of the small bloodvessels, contra-indicates its use in cases of hemorrhage; if not entirely contra-indicated the indication is that it should be used with extreme caution. If faintness from loss of blood demands stimulation, use hot milk or other nutrients easily assimilated. If it is true that alcohol diminishes the coagulating property of the blood, great hesitation should be used in administering it previous to surgical operations, from which severe hemorrhage is anticipated.

In obstetrical cases, demanding a stimulation of the uterus, alcohol has been a favorite remedy. Its utility for this purpose is greatly doubted. I have ceased to use it with this object in view, having frequently witnessed a directly opposite effect from its employment. Instead of exciting uterine contraction, it has allayed it.

Criticism, similar to the above, might be made concerning the use of alcohol in a vast number of diseased conditions. Testimony, from the experience of many eminent physicians, and from carefully prepared statistics, could be adduced, to show the insignificant value of alcohol as a therapeutic agent.

The statistics of the London Temperance Hospital for ten and a half years, show a mortality of 5.05 per cent. The typhoid fever cases had a mortality of less than twelve per cent. The surgical cases numbered 401, and gave a mortality of two per cent. Only three deaths occurred from the 103 major operations. Surely, this is not a bad exhibit for a hospital in the heart of London!

The British Medical Temperance Association has for one of its objects, "to promote investigation as to the action of alcohol in health and disease." Its second annual report gives statistics, gathered from various parts of England, of cases treated without alcohol. The cases presented numbered 1,920, the death-rate of which was 4.06 per cent.

My study of this subject justifies me in formulating the following propositions:

1. Grave responsibility rests upon the medical profession in the use of alcohol as a medicine, on account of its deleterious influence upon the system, and the liability of the patient to contract the habit of using it as a beverage.

2. Alcohol being an acrid narcotic poison, the bottle containing it should be labeled "Poison," as a reminder of this characteristic, and a warning to handle it with care.

3. Alcohol, containing none of the compounds which enter into the construction of the tissues, cannot properly be termed a tissue-forming food.

4. The evidence in favor of the existence of a heat-generating quality in alcohol is not sufficient to warrant the belief that it is a heat-producing food.

5. As a narcotic and anesthetic, alcohol has a limited sphere of adaptation, and is much less valuable than several other narcotics and anesthetics.

6. The stimulating effect of alcohol may be best secured by small doses frequently repeated.

7. From the fact that its stimulating effect results from its paralytic action, alcohol is more properly called a depressant than a stimulant.

8. As an antispasmodic and antiseptic, it may be superseded by other remedies, without detriment to the patient.

9. Although alcohol is a positive antipyretic, and, therefore, useful in the reduction of bodily temperature, it is neither so prompt nor so effective as several other antipyretics.

10. In cases requiring a remedy which will rapidly evaporate, alcohol is useful as an external application.

11. So easy is the acquirement of the alcoholic habit, and so ruinous its consequences to body, mind, and spirit, that extreme caution should be exercised in its use in all cases, and its administration stopped as soon as the desired effect has been secured.

12. Alcohol, as a medicine, should be reserved for emergencies, unusual conditions, and circumstances in which a more reliable and less injurious remedy cannot be obtained.

13. Adulterations of alcoholics is so extensive and so pernicious, and their different preparations so variable in the amount of alcohol they contain, that it is best to demand pure alcohol of a definite strength in medical prescriptions.

14. In the prescription of alcohol, the same care as to exactness of dosage and times of administration should be exercised, as is used in prescribing any other powerful medicine.

15. When intended to act therapeutically, alcoholics should not be prescribed as a beverage and taken *ad libitum*.

16. The fact that methyl alcohol passes very rapidly into and out of the system, is an argument in favor of its more general use for internal administration.

17. So deleterious are the effects of alcohol upon the human body, it is eminently proper to inquire whether its harmfulness does not overbalance its helpfulness, and whether it could not be dropped from our list of therapeutic agents without any serious injury to our patients.

118 Plymouth Avenue.

